# 4-Chloro-*N*′-(2-hydr­oxy-1-naphthyl­idene)benzohydrazide

**DOI:** 10.1107/S1600536808025828

**Published:** 2008-08-16

**Authors:** De-Suo Yang

**Affiliations:** a Department of Chemistry and Chemical Engineering, Baoji University of Arts and Sciences, Baoji 721007, People’s Republic of China

## Abstract

The mol­ecule of the title compound, C_18_H_13_ClN_2_O_2_, displays a *trans* configuration with respect to the C=N double bond. The dihedral angle between the benzene and naphthyl ring systems is 6.0 (2)°. An O—H⋯N hydrogen bond is observed in the mol­ecular structure. In the crystal structure, mol­ecules are linked through inter­molecular N—H⋯O hydrogen bonds and π–π stacking inter­actions [centroid–centroid distance = 3.603 (2) Å], forming chains running along the *b* axis.

## Related literature

For related structures, see: Yang (2006*a* ▶,*b* ▶,*c* ▶,*d* ▶,*e* ▶, 2007*a* ▶,*b* ▶,*c* ▶); Yang & Guo (2006 ▶). For related literature, see: Bernardo *et al.* (1996 ▶); Musie *et al.* (2001 ▶); Paul *et al.* (2002 ▶). For bond-length data, see: Allen *et al.* (1987 ▶).
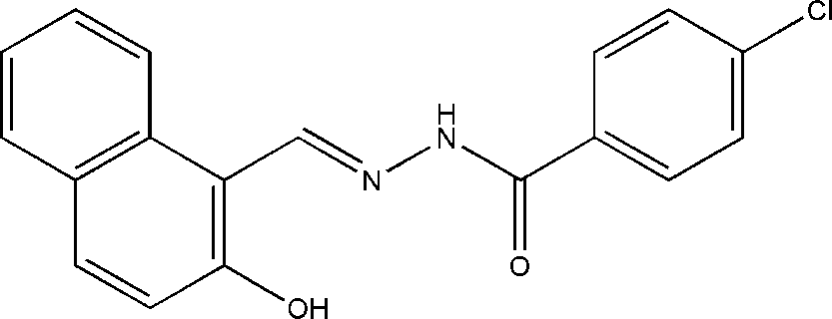


## Experimental

### 

#### Crystal data

C_18_H_13_ClN_2_O_2_
*M* *_r_* = 324.75
Monoclinic, 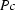

*a* = 6.200 (3) Å
*b* = 4.788 (2) Å
*c* = 25.320 (11) Å
β = 95.844 (7)°
*V* = 747.8 (6) Å^3^
*Z* = 2
Mo *K*α radiation
μ = 0.27 mm^−1^
*T* = 298 (2) K
0.23 × 0.21 × 0.20 mm

#### Data collection

Bruker SMART CCD area-detector diffractometer
Absorption correction: multi-scan (*SADABS*; Sheldrick, 1996 ▶) *T*_min_ = 0.941, *T*_max_ = 0.949
4001 measured reflections
2197 independent reflections
2039 reflections with *I* > 2σ(*I*)
*R*_int_ = 0.017

#### Refinement

*R*[*F* ^2^ > 2σ(*F* ^2^)] = 0.032
*wR*(*F* ^2^) = 0.080
*S* = 1.05
2197 reflections
212 parameters
3 restraints
H atoms treated by a mixture of independent and constrained refinement
Δρ_max_ = 0.12 e Å^−3^
Δρ_min_ = −0.19 e Å^−3^
Absolute structure: Flack (1983 ▶), with 596 Friedel pairs
Flack parameter: 0.03 (7)

### 

Data collection: *SMART* (Bruker, 2002 ▶); cell refinement: *SAINT* (Bruker, 2002 ▶); data reduction: *SAINT* ; program(s) used to solve structure: *SHELXS97* (Sheldrick, 2008 ▶); program(s) used to refine structure: *SHELXL97* (Sheldrick, 2008 ▶); molecular graphics: *SHELXTL* (Sheldrick, 2008 ▶); software used to prepare material for publication: *SHELXL97*.

## Supplementary Material

Crystal structure: contains datablocks global, I. DOI: 10.1107/S1600536808025828/ci2654sup1.cif

Structure factors: contains datablocks I. DOI: 10.1107/S1600536808025828/ci2654Isup2.hkl

Additional supplementary materials:  crystallographic information; 3D view; checkCIF report

## Figures and Tables

**Table 1:** Hydrogen-bond geometry (Å, °)

*D*—H⋯*A*	*D*—H	H⋯*A*	*D*⋯*A*	*D*—H⋯*A*
O1—H1⋯N1	0.82	1.87	2.584 (2)	145
N2—H2⋯O2^i^	0.90 (1)	1.98 (1)	2.858 (3)	165 (4)

Symmetry code: (i) 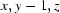.

**Fig. 1.**
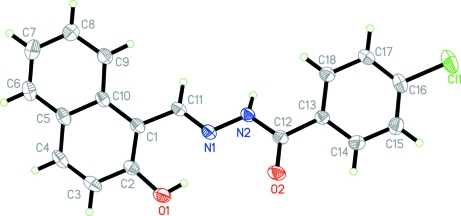
The molecular structure of the title compound, showing the atom-numbering scheme. Displacement ellipsoids are drawn at the 30% probability level.

**Fig. 2.**
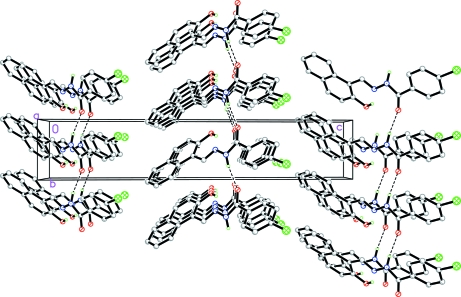
Crystal packing packing of the title compound, viewed along the a axis. H atoms not involved in hydrogen bonding (dashed lines) have been omitted for clarity.

## supplementary crystallographic information

### Comment

Schiff base compounds have been of great interest for a long time. These compounds play an important role in the development of coordination chemistry (Musie *et al.*, 2001; Bernardo *et al.*, 1996; Paul *et al.*, 2002). Recently, we have reported crystal structures of a few Schiff base compounds (Yang, 2006*a*,*b*,*c*,*d*,*e*, 2007*a*,*b*,*c*; Yang & Guo, 2006). As a further investigation of this work, the crystal structure of the title compound is reported here.

The molecule of the title compound displays a *trans* configuration with respect to the C═N double bond (Fig. 1). The dihedral angle between the benzene and naphthyl rings is 6.0 (2)°. All the bond lengths are within normal ranges (Allen *et al.*, 1987). The C11═N1 bond length of 1.275 (3) Å conforms to the value for a double bond. The bond length of 1.353 (3) Å between atoms C12 and N2 is intermediate between an N—N single bond and an N═N double bond, because of conjugation effects in the molecule. An intramolecular O—H···N hydrogen bond is observed.

In the crystal structure, molecules are linked through intermolecular N—H···O hydrogen bonds (Table 1), forming chains running along the *b* axis (Fig. 2). The chain is strengthened by π–π interactions between C1–C5/C10 and C5–C10 benzene rings (centroid–centroid distance is 3.603 (2) Å).

### Experimental

2-Hydroxy-1-naphthylaldehyde (0.1 mmol, 17.2 mg) and 4-chlorobenzohydrazide (0.1 mmol, 17.0 mg) were dissolved in methanol (10 ml). The mixture was stirred at room temperature to give a clear colourless solution. Single crystals of the title compound were obtained by gradual evaporation of the solvent over a period of 8 d at room temperature.

### Refinement

Atom H2 was located in a difference Fourier map and refined isotropically, with the N—H distance restrained to 0.90 (1) Å. Other H atoms were placed in idealized positions and constrained to ride on their parent atoms, with O—H = 0.82 Å, C—H = 0.93 Å and *U*_iso_(H) = 1.2*U*_eq_(C) and 1.5*U*_eq_(O).

### Crystal data

**Table:**

C_18_H_13_ClN_2_O_2_	*F*_000_ = 336
*M**_r_* = 324.75	*D*_x_ = 1.442 Mg m^−^^3^
Monoclinic, *P**c*	Mo *K*α radiation λ = 0.71073 Å
Hall symbol: P -2yc	Cell parameters from 2145 reflections
*a* = 6.200 (3) Å	θ = 2.8–29.3º
*b* = 4.788 (2) Å	µ = 0.27 mm^−^^1^
*c* = 25.320 (11) Å	*T* = 298 (2) K
β = 95.844 (7)º	Block, colourless
*V* = 747.8 (6) Å^3^	0.23 × 0.21 × 0.20 mm
*Z* = 2	

### Data collection

**Table:**

Bruker SMART CCD area-detector diffractometer	2197 independent reflections
Radiation source: fine-focus sealed tube	2039 reflections with *I* > 2σ(*I*)
Monochromator: graphite	*R*_int_ = 0.017
*T* = 298(2) K	θ_max_ = 27.0º
ω scans	θ_min_ = 3.2º
Absorption correction: multi-scan(SADABS; Sheldrick, 1996)	*h* = −5→7
*T*_min_ = 0.941, *T*_max_ = 0.949	*k* = −6→6
4001 measured reflections	*l* = −31→26

### Refinement

**Table:**

Refinement on *F*^2^	Hydrogen site location: inferred from neighbouring sites
Least-squares matrix: full	H atoms treated by a mixture of independent and constrained refinement
*R*[*F*^2^ > 2σ(*F*^2^)] = 0.032	*w* = 1/[σ^2^(*F*_o_^2^) + (0.0403*P*)^2^ + 0.1026*P*] where *P* = (*F*_o_^2^ + 2*F*_c_^2^)/3
*wR*(*F*^2^) = 0.080	(Δ/σ)_max_ = 0.001
*S* = 1.05	Δρ_max_ = 0.12 e Å^−^^3^
2197 reflections	Δρ_min_ = −0.19 e Å^−^^3^
212 parameters	Extinction correction: none
3 restraints	Absolute structure: Flack (1983), with 596 Friedel pairs
Primary atom site location: structure-invariant direct methods	Flack parameter: 0.03 (7)
Secondary atom site location: difference Fourier map	

### Special details

**Table:**

Geometry. All e.s.d.'s (except the e.s.d. in the dihedral angle between two l.s. planes) are estimated using the full covariance matrix. The cell e.s.d.'s are taken into account individually in the estimation of e.s.d.'s in distances, angles and torsion angles; correlations between e.s.d.'s in cell parameters are only used when they are defined by crystal symmetry. An approximate (isotropic) treatment of cell e.s.d.'s is used for estimating e.s.d.'s involving l.s. planes.
Refinement. Refinement of *F*^2^ against ALL reflections. The weighted *R*-factor *wR* and goodness of fit *S* are based on *F*^2^, conventional *R*-factors *R* are based on *F*, with *F* set to zero for negative *F*^2^. The threshold expression of *F*^2^ > σ(*F*^2^) is used only for calculating *R*-factors(gt) *etc*. and is not relevant to the choice of reflections for refinement. *R*-factors based on *F*^2^ are statistically about twice as large as those based on *F*, and *R*- factors based on ALL data will be even larger.

### Fractional atomic coordinates and isotropic or equivalent isotropic displacement parameters (Å^2^)

**Table:**

	*x*	*y*	*z*	*U*_iso_*/*U*_eq_	
Cl1	1.44733 (12)	0.28331 (19)	0.25960 (4)	0.0780 (3)	
N1	0.3640 (3)	0.4990 (4)	0.05683 (7)	0.0395 (4)	
N2	0.5480 (3)	0.4210 (4)	0.08892 (8)	0.0390 (4)	
O1	0.0022 (3)	0.7705 (3)	0.04417 (7)	0.0512 (4)	
H1	0.1220	0.7293	0.0589	0.077*	
O2	0.5730 (3)	0.8546 (3)	0.12464 (7)	0.0504 (4)	
C1	0.0931 (3)	0.3955 (4)	−0.01348 (9)	0.0378 (5)	
C2	−0.0450 (3)	0.6040 (4)	0.00131 (9)	0.0404 (5)	
C3	−0.2478 (4)	0.6510 (5)	−0.02775 (11)	0.0515 (6)	
H3	−0.3413	0.7847	−0.0163	0.062*	
C4	−0.3071 (4)	0.5020 (5)	−0.07244 (11)	0.0529 (6)	
H4	−0.4422	0.5341	−0.0909	0.063*	
C5	−0.1684 (4)	0.2987 (5)	−0.09167 (10)	0.0458 (5)	
C6	−0.2251 (4)	0.1535 (5)	−0.13960 (10)	0.0544 (6)	
H6	−0.3580	0.1895	−0.1589	0.065*	
C7	−0.0893 (5)	−0.0380 (6)	−0.15817 (10)	0.0587 (7)	
H7	−0.1289	−0.1318	−0.1898	0.070*	
C8	0.1105 (5)	−0.0923 (6)	−0.12918 (10)	0.0549 (6)	
H8	0.2035	−0.2233	−0.1418	0.066*	
C9	0.1709 (4)	0.0441 (5)	−0.08267 (9)	0.0478 (6)	
H9	0.3051	0.0049	−0.0643	0.057*	
C10	0.0348 (3)	0.2428 (5)	−0.06184 (9)	0.0384 (5)	
C11	0.2922 (3)	0.3316 (5)	0.01999 (9)	0.0393 (5)	
H11	0.3673	0.1678	0.0145	0.047*	
C12	0.6429 (3)	0.6152 (4)	0.12246 (8)	0.0371 (5)	
C13	0.8398 (3)	0.5189 (4)	0.15662 (8)	0.0357 (4)	
C14	0.8939 (4)	0.6535 (5)	0.20461 (10)	0.0468 (6)	
H14	0.8045	0.7941	0.2153	0.056*	
C15	1.0795 (4)	0.5809 (6)	0.23675 (10)	0.0532 (6)	
H15	1.1155	0.6701	0.2691	0.064*	
C16	1.2100 (4)	0.3728 (5)	0.21960 (9)	0.0483 (6)	
C17	1.1626 (4)	0.2370 (5)	0.17207 (10)	0.0449 (5)	
H17	1.2543	0.0991	0.1613	0.054*	
C18	0.9751 (3)	0.3101 (4)	0.14053 (9)	0.0407 (5)	
H18	0.9393	0.2189	0.1084	0.049*	
H2	0.576 (6)	0.240 (3)	0.0963 (14)	0.080*	

### Atomic displacement parameters (Å^2^)

**Table:**

	*U*^11^	*U*^22^	*U*^33^	*U*^12^	*U*^13^	*U*^23^
Cl1	0.0468 (3)	0.1144 (6)	0.0692 (4)	0.0089 (4)	−0.0112 (3)	0.0192 (4)
N1	0.0344 (9)	0.0357 (9)	0.0476 (10)	0.0038 (7)	0.0003 (8)	0.0045 (7)
N2	0.0373 (9)	0.0305 (8)	0.0475 (10)	0.0053 (8)	−0.0031 (8)	0.0022 (7)
O1	0.0545 (10)	0.0446 (9)	0.0552 (10)	0.0141 (8)	0.0086 (8)	0.0030 (8)
O2	0.0490 (9)	0.0290 (7)	0.0715 (11)	0.0077 (7)	−0.0016 (8)	−0.0015 (7)
C1	0.0326 (11)	0.0347 (10)	0.0460 (12)	0.0008 (9)	0.0040 (9)	0.0108 (9)
C2	0.0382 (12)	0.0373 (12)	0.0465 (12)	0.0038 (9)	0.0086 (9)	0.0103 (9)
C3	0.0400 (12)	0.0496 (14)	0.0662 (16)	0.0121 (11)	0.0114 (11)	0.0140 (12)
C4	0.0336 (12)	0.0531 (14)	0.0700 (17)	0.0073 (10)	−0.0036 (11)	0.0165 (12)
C5	0.0367 (12)	0.0431 (12)	0.0561 (14)	−0.0036 (10)	−0.0017 (10)	0.0164 (10)
C6	0.0490 (14)	0.0546 (15)	0.0558 (15)	−0.0049 (12)	−0.0129 (11)	0.0132 (12)
C7	0.0672 (17)	0.0599 (16)	0.0468 (15)	−0.0122 (14)	−0.0056 (12)	0.0016 (12)
C8	0.0576 (14)	0.0563 (15)	0.0507 (15)	0.0031 (12)	0.0050 (11)	−0.0020 (12)
C9	0.0427 (13)	0.0486 (13)	0.0512 (14)	0.0048 (10)	0.0008 (10)	0.0022 (10)
C10	0.0330 (10)	0.0350 (12)	0.0466 (12)	−0.0017 (8)	0.0014 (8)	0.0102 (8)
C11	0.0345 (11)	0.0343 (10)	0.0491 (12)	0.0053 (8)	0.0038 (9)	0.0047 (9)
C12	0.0367 (11)	0.0311 (10)	0.0439 (12)	0.0030 (9)	0.0059 (9)	0.0046 (8)
C13	0.0371 (11)	0.0283 (10)	0.0416 (11)	0.0005 (8)	0.0030 (8)	0.0041 (8)
C14	0.0503 (14)	0.0391 (12)	0.0510 (14)	0.0056 (10)	0.0053 (11)	−0.0025 (10)
C15	0.0540 (15)	0.0598 (16)	0.0443 (14)	−0.0019 (12)	−0.0028 (11)	−0.0037 (11)
C16	0.0352 (11)	0.0594 (15)	0.0489 (14)	−0.0019 (11)	−0.0020 (10)	0.0163 (11)
C17	0.0371 (12)	0.0464 (13)	0.0518 (14)	0.0093 (10)	0.0074 (10)	0.0080 (10)
C18	0.0398 (12)	0.0373 (11)	0.0453 (12)	0.0029 (9)	0.0051 (9)	0.0022 (9)

### Geometric parameters (Å, °)

**Table:**

Cl1—C16	1.752 (2)	C6—H6	0.93
N1—C11	1.275 (3)	C7—C8	1.398 (4)
N1—N2	1.383 (2)	C7—H7	0.93
N2—C12	1.353 (3)	C8—C9	1.365 (3)
N2—H2	0.899 (10)	C8—H8	0.93
O1—C2	1.354 (3)	C9—C10	1.410 (3)
O1—H1	0.82	C9—H9	0.93
O2—C12	1.228 (2)	C11—H11	0.93
C1—C2	1.392 (3)	C12—C13	1.495 (3)
C1—C10	1.440 (3)	C13—C14	1.386 (3)
C1—C11	1.457 (3)	C13—C18	1.392 (3)
C2—C3	1.409 (3)	C14—C15	1.385 (3)
C3—C4	1.356 (4)	C14—H14	0.93
C3—H3	0.93	C15—C16	1.381 (4)
C4—C5	1.418 (4)	C15—H15	0.93
C4—H4	0.93	C16—C17	1.373 (3)
C5—C6	1.412 (4)	C17—C18	1.387 (3)
C5—C10	1.427 (3)	C17—H17	0.93
C6—C7	1.361 (4)	C18—H18	0.93
			
C11—N1—N2	117.76 (18)	C8—C9—H9	119.2
C12—N2—N1	117.57 (16)	C10—C9—H9	119.2
C12—N2—H2	118 (2)	C9—C10—C5	117.3 (2)
N1—N2—H2	121 (2)	C9—C10—C1	123.55 (19)
C2—O1—H1	109.5	C5—C10—C1	119.1 (2)
C2—C1—C10	119.01 (18)	N1—C11—C1	120.4 (2)
C2—C1—C11	120.14 (19)	N1—C11—H11	119.8
C10—C1—C11	120.85 (19)	C1—C11—H11	119.8
O1—C2—C1	123.34 (19)	O2—C12—N2	122.48 (19)
O1—C2—C3	115.7 (2)	O2—C12—C13	122.18 (19)
C1—C2—C3	121.0 (2)	N2—C12—C13	115.34 (16)
C4—C3—C2	120.2 (2)	C14—C13—C18	119.4 (2)
C4—C3—H3	119.9	C14—C13—C12	118.30 (19)
C2—C3—H3	119.9	C18—C13—C12	122.20 (19)
C3—C4—C5	121.7 (2)	C15—C14—C13	120.7 (2)
C3—C4—H4	119.2	C15—C14—H14	119.6
C5—C4—H4	119.2	C13—C14—H14	119.6
C6—C5—C4	121.8 (2)	C16—C15—C14	118.3 (2)
C6—C5—C10	119.5 (2)	C16—C15—H15	120.8
C4—C5—C10	118.7 (2)	C14—C15—H15	120.8
C7—C6—C5	121.3 (2)	C17—C16—C15	122.5 (2)
C7—C6—H6	119.3	C17—C16—Cl1	118.9 (2)
C5—C6—H6	119.3	C15—C16—Cl1	118.62 (19)
C6—C7—C8	119.4 (2)	C16—C17—C18	118.5 (2)
C6—C7—H7	120.3	C16—C17—H17	120.7
C8—C7—H7	120.3	C18—C17—H17	120.7
C9—C8—C7	121.0 (3)	C17—C18—C13	120.5 (2)
C9—C8—H8	119.5	C17—C18—H18	119.8
C7—C8—H8	119.5	C13—C18—H18	119.8
C8—C9—C10	121.6 (2)		

### Hydrogen-bond geometry (Å, °)

**Table:**

*D*—H···*A*	*D*—H	H···*A*	*D*···*A*	*D*—H···*A*
O1—H1···N1	0.82	1.87	2.584 (2)	145
N2—H2···O2^i^	0.90 (1)	1.98 (1)	2.858 (3)	165 (4)

Symmetry codes: (i) *x*, *y*−1, *z*.
